# 822. Is the Juice Worth the Squeeze: Ophthalmologist Examination for All Hospitalized Patients with Candidemia in a Rural Community

**DOI:** 10.1093/ofid/ofad500.867

**Published:** 2023-11-27

**Authors:** Hyun Sue Kim, Anthony Baffoe-Bonnie

**Affiliations:** Virginia Tech Carilion School of Medicine, Roanoke, Virginia; Carilion Clinic, Roanoke, Virginia

## Abstract

**Background:**

Candidemia is a common cause of bloodstream infections in hospitalized patients in the United States. Ocular manifestations of candidemia include chorioretinitis and endophthalmitis, which can lead to the loss of vision. Currently, there are opposing recommendations on whether all patients with candidemia should have a dilated ophthalmologic examination. We describe a rural patient population with candidemia who underwent an ophthalmology consultation and the associated clinical features and risk factors for an abnormal eye exam. We analyze the impact of ophthalmology consults on the treatment, management, and outcome.

**Methods:**

We conducted a retrospective cross-sectional study of consecutive candidemia cases from 2015 to 2023. We abstracted patient demographics, clinical, and microbiologic data.

**Results:**

We found that 218 candidemia patients with an average age of 54 years (range, 5-94) underwent an ophthalmologic evaluation. The average duration between hospital admission and consultation was 12 days. 24% of patients reported visual symptoms at the time of the consult and of those, 49% reported decreased visual acuity. Age, insurance status, and the number of days between hospital admission and consultation were strongly associated with positive findings.

**Conclusion:**

Abnormal eye findings were not uncommon and were seen more frequently in certain demographic groups. In resource-limited settings, a more nuanced consultation strategy informed by local data may be reasonable.

**Disclosures:**

**All Authors**: No reported disclosures

